# Structural mapping of NTCP distinguishes its dual functionality as a hepatitis B virus receptor and bile acid transporter

**DOI:** 10.1371/journal.ppat.1013824

**Published:** 2026-01-16

**Authors:** Kayo Matsuzawa, Toru Ekimoto, Chisa Kobayashi, Kaho Shionoya, Junki Mifune, Takeshi Morita, Junko S. Takeuchi, Sam-Yong Park, Mitsunori Ikeguchi, Camille Sureau, Atsushi Kawaguchi, Koichi Watashi

**Affiliations:** 1 Department of Drug Development, National Institute of Infectious Diseases, Japan Institute for Health Security, Tokyo, Japan; 2 Department of Infection Biology, Transborder Medical Research Center, Institute of Medicine, University of Tsukuba, Tsukuba, Japan; 3 Computational Life Science Laboratory, Graduate School of Medical Life Science, Yokohama City University, Yokohama, Japan; 4 Department of Academic-Industrial Partnerships Promotion, Center for Clinical Sciences, Japan Institute for Health Security, Tokyo, Japan; 5 Drug Design Laboratory, Graduate School of Medical Life Science, Yokohama City University, Yokohama, Japan; 6 HPC- and AI-driven Drug Development Platform Division, Center for Computational Science, RIKEN, Yokohama, Japan; 7 INSERM U1259, Université de Tours, Tours, France; 8 MIRAI, Japan Science and Technology Agency, Saitama, Japan; Pennsylvania State University College of Medicine: Penn State College of Medicine, UNITED STATES OF AMERICA

## Abstract

Sodium taurocholate cotransporting polypeptide (NTCP) is a hepatic transmembrane (TM) protein that functions both as a bile acid transporter and as a host receptor for hepatitis B and D viruses via the viral preS1 binding. The structural and mechanistical determinants for NTCP’s dual functions remain largely undefined. In this study, using comprehensive structure-guided alanine-scanning mutagenesis based on the cryo-electron microscopy structure of the preS1/NTCP complex, we identified 13, 8 and 9 NTCP amino acid residues critical for viral infection, preS1 binding, and bile acid transport, respectively. Key residues overlappingly regulating viral receptor and transporter functions were located primarily at TM1 and TM8, whereas TM5 and outer-surface NTCP loops mediated viral receptor-specific activity. In addition to 8 amino acids key to preS1 binding, 5 residues likely acted at a post-preS1 binding step of infection. We further found naturally-occurring single nucleotide polymorphism-associated F274C/S NTCP variants abolished viral receptor function, via the potential conformational changes in bile acid tunnel and outer-surface hollow, as analyzed by molecular dynamics simulations. Our domain-specific structural-functional map of NTCP defines the mechanism how NTCP’s dual functionality is separately regulated, and provides a framework for designing selective antiviral agents that preserve bile acid transport.

## Author summary

Sodium taurocholate cotransporting polypeptide (NTCP) has dual functions, as a bile acid transporter and as an entry receptor for hepatitis B and D viruses. It remains unknown whether the two functions are mediated by the same or different mechanism. This study clarify the molecular determinants in NTCP for the viral receptor and bile acid transporter functions by mutation analysis based on the cryo-electron microscopy structure. We identified the amino acids in NTCP responsible for supporting viral binding, infection, bile acid transport, and cell surface protein production, and found that those involved in virus infection showed a partially overlapping but entirely distinct map from those related with bile acid transport. From this profile, we also revealed a single nucleotide polymorphism causing the F274C/S mutation in NTCP as a variant showing less susceptible to infection. Our analysis disclose the basic information how NTCP exerts the dual functionality for hepatitis B and D virus infection and bile acid transport.

## Introduction

Virus-host receptor interaction is a key to define the fundamental viral characteristics, including transmission efficiency, host range, tissue tropism, and pathogenesis, thus serving as an attractive target for the development of antiviral agents [1]. Hepatitis B virus (HBV) displays three viral surface proteins, large, middle, and small proteins at the surface of infectious particles. Among them, the N-terminal region of the large surface protein, called the preS1 region, especially its 2–48 amino acid segment [preS1(2–48)], plays a crucial role in binding to the host entry receptor, sodium taurocholate cotransporting polypeptide (NTCP) [2–5]. Following a non-specific and low affinity binding to host heparan sulfate proteoglycans, HBV recognizes host cells by more specific and high affinity binding of preS1(2–48) to NTCP [6]. The preS1-NTCP interaction triggers internalization of HBV with involving many other host factors that mediate membrane trafficking to an intracellular compartment in which fusion of viral envelope with cellular membrane are likely to occur. The viral nucleocapsid released in the cytoplasm translocates into the nucleus to eventually form covalently closed circular DNA (cccDNA), a template for virus replication [6]. Hepatitis D virus (HDV), a HBV satellite virus, bears the HBV-derived surface proteins that ensures the same function at viral early entry. HDV is thus often used as a model for analyzing the HBV early entry mechanism [6].

The HBV/HDV entry receptor, NTCP, encoded by the *solute carrier family 10A1 (SLC10A1)* gene, is specifically expressed at the surface of hepatocytes and originally functions as a transporter for bile acid uptake together with sodium ions from the blood to hepatocytes [6]. A *SLC10A1* single nucleotide polymorphism (SNP), rs2296651, the most frequent variant present in 3–10% of the East Asian population, leads to a serin-to-phenylalanine substitution at position 267 (S267F) in NTCP and to a loss of bile acid transport function that results in a drastic elevation in serum bile acid levels [7,8]. This NTCP S267F-associated SNP population showed a significantly lower frequency in developing chronic hepatitis B and the related hepatocellular carcinoma, demonstrating the crucial role of NTCP in HBV infection and pathogenesis [9,10]. These evidences highlight the dual role of NTCP as a viral receptor, and bile acid transporter, and its functional diversity by sequence substitutions. Evolutionally, the transporter function of NTCP is conserved among a wide range of species, at least from fishes to humans, whereas the HBV receptor function is far more species specific, being functionally deficient in NTCP derived from most non-human species including Old World Monkeys, pigs, rats, and mice [11–13]. Such a clear functional contrast implies that the two functions of NTCP are regulated independently. However, previous molecular biological analyses reported that i) the viral receptor function was impaired by introduction of transporter-deficient mutations in NTCP, and ii) the mutual functional competition occurred between the preS1(2–48) peptide and bile acids [14–16], suggesting an overlapping regulation of the two functions. With only these fragmented reports, the full picture of how the two functions are regulated in NTCP either independently or overlappingly remains unclear.

**Fig 1:**
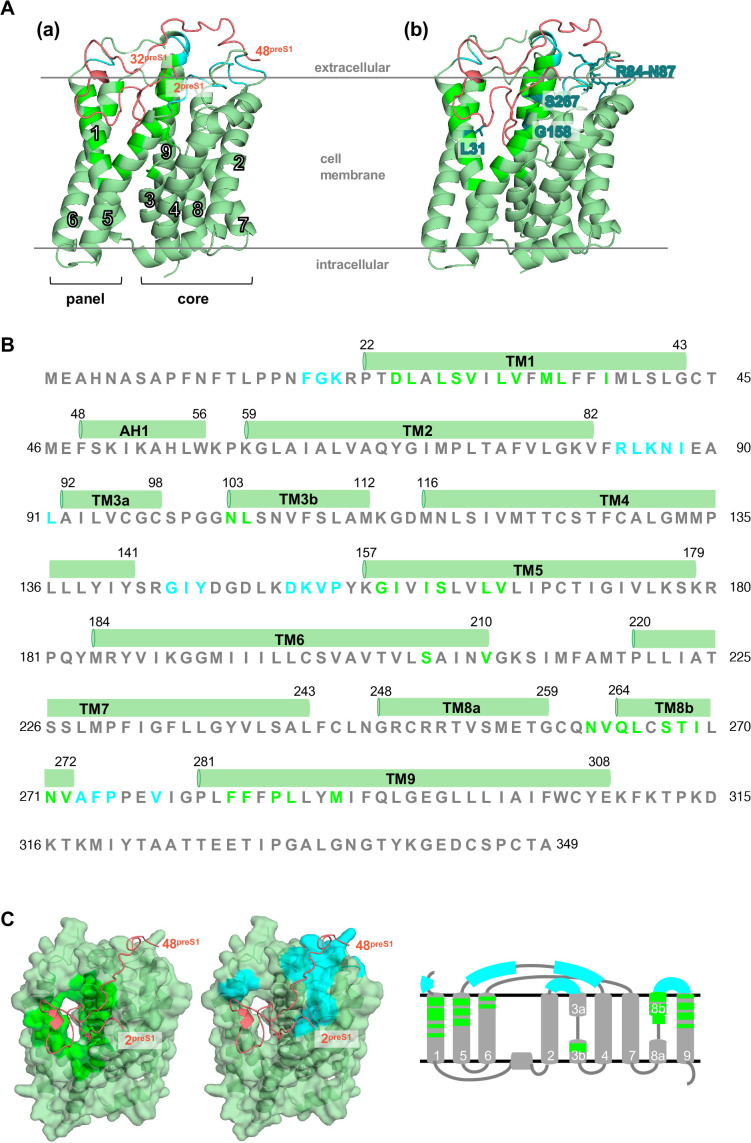
54 amino acids in NTCP closely contacting with the 2–48 aa of preS1. **(A)** Structure of NTCP in complex with 2–48 aa of preS1 [preS1(2–48)] (PDB: 8HRY). NTCP and preS1(2–48) are colored in pale green and pink, respectively. 54 amino acids resided within 4.5 Å of preS1 amino acids are colored in bright green (facing the bile acid tunnel) or cyan (facing the extracellular surface). (a) White numbers indicate the transmembrane (TM) helix number of NTCP. The panel domain consists of TM1, 5, and 6; the core domain consists of TM2, 3, 4, 7, 8, and 9. (b) NTCP amino acids reportedly important for supporting preS1 binding and HBV/HDV infection, L31, R84-N87, G158, and S267, are shown by turquoise. **(B)** Amino acid sequence of NTCP. 54 amino acids closely contacting with preS1(2–48) are colored in bright green and cyan, as indicated in (A). The TM numbers are shown above the sequence. Amino acid numbers are also indicated. **(C)** Transparent surface structures (left) and diagram of TM structures of NTCP (right). 54 amino acids are shown in bright green (facing the bile acid tunnel) and cyan (facing the extracellular surface).

Recently, we and other groups solved the cryo-electron microscopy (cryo-EM) structure of apo-state NTCP and its complex form with preS1(2–48) [17–20]. NTCP has nine transmembrane (TM) helices constituting the two domains, the panel domain (TM1, 5, and 6) and the core domain (TM2, 3, 4, 7, 8, and 9). These two domains form a hydrophobic tunnel between these that functions as a pore for bile acid uptake. In the preS1(2–48)/ NTCP complex structure, TM1, 3, 5, 6, 8, and 9 facing on the tunnel closely contact to the N-terminal part of preS1(2–48), and the extracellular outer-surface area including TM2–3, TM4–5, and TM8–9 loops contact to the C-terminal region of preS1(2–48) (Fig 1A) [21,22]. This large interface is regarded to contribute to the high affinity interaction with preS1. So far, NTCP amino acid positions 158, 84–87, 267, and 146 resided at wide areas in NTCP from the tunnel to the extracellular surface are reported to be important for HBV infection [12,23–27], however, the overall role of the NTCP structural segments within the large interface in the viral receptor function remains largely undefined. Given that NTCP is a prominent antiviral target, as demonstrated by the development of Myrcludex-B (also known as Bulevirtide) as a clinically-approved anti-HDV agent [6,28], such information would be critical for future design of new antiviral agents.

In this study, we conducted comprehensive mutagenesis analysis based on the structural information and examined the NTCP mutant activities for preS1 binding, viral infection, and bile acid uptake. We could discriminate between amino acids essential for the viral receptor function and those involved in the bile acid uptake to depict the functional map of the NTCP protein. In addition, we analyzed the impact of SNP-associated amino acid substitutions in NTCP on the viral receptor function using infection assay and molecular dynamics simulation for understanding the mechanism of functional alteration. This study provides basic information to understand the dual functionality of NTCP that is useful for the development of selective antiviral agents that preserve the bile acid transport.

## Results

### Selection of NTCP amino acids closely contacting with preS1

The cryo-EM structure of preS1(2–48)/NTCP complex (PDB: 8HRY) we have recently reported shows that the N-terminal 30 amino acid residues of preS1 are inserted into the bile acid tunnel of NTCP, and the C-terminal stretch of preS1(2–48) crawls along with the extracellular surface of NTCP (Fig 1Aa) [21,22]. To comprehensively identify NTCP amino acids involved in preS1 binding, we focused on the amino acids closely contacting with preS1: From the 302 amino acids solved in the cryo-EM structure (11-312 aa in NTCP), we selected those located within 4.5 Å from any of the preS1 amino acids (Table 1), based on a distance-based, unbiased criterion to capture both the tunnel-embedded N-terminal and the surface-tracking C-terminal interfaces of preS1, and found 54 amino acids, including 34 facing the bile acid tunnel (Fig 1A–1C, bright green) and 20 on the extracellular surface (Fig 1A–1C, cyan). These 34 amino acids contain 10 amino acids in TM1, two in TM3b, six in TM5, two in TM6, nine in TM8, and five in TM9 on the bile acid tunnel, and 20 amino acids include three from N-terminus to TM1, six in the TM2–3 loop, seven in the TM4–5 loop, and four in the TM8b-9 loop that are resided on the extracellular surface (Fig 1B and 1C). These amino acids contain those reportedly important for the viral receptor function, L31, G158, R84-N87, and S267 (Fig 1Ab, turquoise) [12,14,15,19,23–26].

**Table 1:** The pairs of 54 amino acid residues in NTCP and their counterpart preS1 amino acids located within 4.5 Å in distance.

NTCP	preS1	Distance (Å)	NTCP	preS1	Distance (Å)	NTCP	preS1	Distance (Å)	NTCP	preS1	distance (Å)
F18	F14	3.93 Å	I38	L11	4.45 Å	G158	S6	3.39 Å	I269	N4	4.23 Å
G19	N26	2.65 Å	R84	V47	3.99 Å		V7	3.89 Å	N271	P15	4.31 Å
	D16	4.35 Å	L85	V47	3.67 Å		P8	3.24 Å		W32	4.00 Å
K20	N26	3.67 Å	K86	A44	3.99 Å	I159	P8	3.99 Å		K38	3.53 Å
	S27	3.35 Å		N45	3.49 Å	I161	V7	3.68 Å	V272	T3	3.94 Å
	E28	4.20 Å		K46	4.34 Å	S162	V7	3.96 Å		N4	3.80 Å
	P30	4.27 Å		V47	4.03 Å		P8	3.28 Å		W32	3.58 Å
D24	D16	4.16 Å	N87	W41	3.26 Å		L19	4.07 Å		D33	3.65 Å
	N26	3.61 Å		D43	3.15 Å	L165	L19	4.03 Å		N35	3.06 Å
L25	F23	3.73 Å		A44	3.22 Å	V166	P10	4.20 Å		K38	3.00 Å
	N26	3.46 Å		K46	2.88 Å		L19	4.24 Å	A273	N35	4.21 Å
L27	F14	4.38 Å		V47	4.16 Å	S206	F13	3.61 Å		K38	3.69 Å
	H17	3.27 Å		G48	3.23 Å	V210	F14	4.01 Å		D39	3.22 Å
S28	D16	3.68 Å	I88	W41	3.53 Å	N262	N9	3.66 Å	F274	K38	3.88 Å
	H17	4.02 Å		A44	2.84 Å		P10	3.58 Å		D39	3.17 Å
	Q18	4.12 Å		N45	3.59 Å	V263	G12	3.39 Å		W41	3.56 Å
	F23	3.64 Å	L91	W41	3.85 Å	Q264	S6	4.47 Å	P275	K38	4.05 Å
	N26	3.78 Å	N103	P10	3.42 Å		P8	3.49 Å		D39	3.23 Å
V29	F23	3.63 Å	L104	L11	4.09 Å		N9	3.45 Å		N40	3.35 Å
L31	N9	3.73 Å	G144	G48	4.41 Å		G12	3.44 Å	V278	N40	3.50 Å
	L11	3.24 Å	I145	W41	4.26 Å		F13	4.39 Å		A44	3.89 Å
	G12	4.28 Å	Y146	D39	3.21 Å		F14	2.44 Å	F283	F14	4.21 Å
	F13	4.43 Å		W41	3.38 Å		P15	3.30 Å		P15	3.86 Å
	H17	3.45 Å	D152	G2	3.75 Å		D16	4.34 Å	F284	F14	4.05 Å
V32	N9	4.25 Å		T3	3.29 Å		H17	2.88 Å	P286	F13	3.47 Å
	Q18	3.54 Å		N4	4.27 Å	L265	P8	3.53 Å	L287	F13	3.81 Å
	L19	4.11 Å	K153	T3	4.22 Å	S267	F13	4.05 Å		F14	4.43 Å
	F23	4.02 Å		N4	2.98 Å		F14	3.60 Å	M290	P10	4.11 Å
M34	L11	4.09 Å		N35	3.62 Å		P15	3.39 Å		L11	4.32 Å
L35	N9	3.72 Å	V154	N4	4.43 Å	T268	N4	3.91 Å		F13	4.03 Å
	P10	4.40 Å	P155	N4	3.51 Å		S6	3.59 Å			
	L11	3.69 Å		S6	3.77 Å		P15	4.15 Å			
							W32	4.06 Å			

We then assessed whether these 54 amino acids are involved in 1) supporting viral infection, 2) mediating preS1 binding, and 3) bile acid uptake, as well as 4) cell surface expression in the following analysis: We constructed the plasmids encoding the NTCP carrying an alanine mutation at these 54 amino acids and overexpressed these NTCP mutants or the wild type in HepG2 cells (which do not originally express endogenous NTCP) to document the functions listed above.

### Capacity of 54 NTCP mutants for supporting viral infection

**Fig 2:**
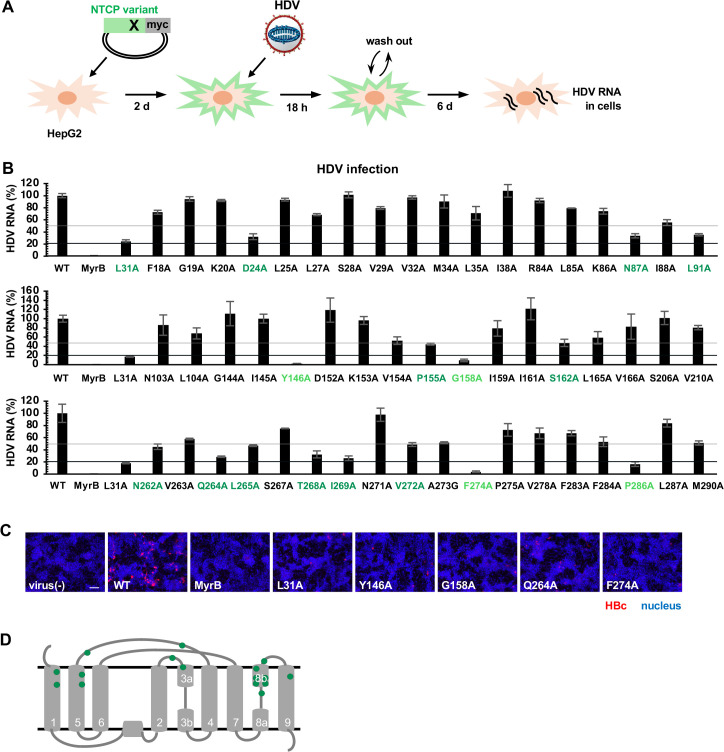
Capacity of 54 NTCP mutants to support viral infection. **(A)** Schematic representation of the HDV infection assay in NTCP mutant-overexpressing cells. HepG2 cells were transfected with the plasmids encoding the NTCP wild type or the 54 alanine mutants for two days, and were then inoculated with HDV for 18 h to detect intracellular HDV RNA at 7 days post-inoculation by real time RT-PCR. **(B)** Quantified intracellular HDV RNA levels are shown by setting that in cells overexpressing the wild type NTCP (WT) as 100%. Myrcludex-B (MyrB), an HBV/HDV entry inhibitor, was used as a positive control to reduce viral infection in cells overexpressing WT. The NTCP mutant at L31 (L31A) was also used as a positive control that shows a reduced activity. Gray and black lines indicate 50% and 20% of the signal for WT, respectively. Mutants indicating 20-50% and less than 20% are shown in green and light green, respectively. **(C)** HBV infection assay for the representative NTCP mutants was performed with a similar procedure to that shown in (A) at 13 days post-inoculation to detect intracellular HBc antigen by immunofluorescence analysis. Scale bar, 100 μm. **(D)** Positions of 16 amino acids showing the signals less than 50% of the control by alanine mutation are shown in green in the TM structure diagram.

To evaluate the capacity of 54 NTCP mutants for supporting NTCP-dependent viral infection, we employed HDV infection assay, which, in our hands, is faster in assay period and more sensitive and reproducible than HBV infection assay, as advantages especially for evaluating large number of samples side by side. We transfected HepG2 cells with the expression plasmid for the wild type (WT) and the mutant NTCPs having an alanine mutation and then exposed to HDV for 18 h, followed by washing free virus and culturing the cells for additional six days to quantify intracellular HDV RNA as a marker of infection (Fig 2A). As an experimental control, treatment of WT-carrying cells with an entry inhibitor, Myrcludex-B, dramatically reduced HDV RNA to below 1% of the untreated cells (Fig 2B, MyrB). In addition, the alanine mutant at L31, reported to be important for HBV infection [19] used as a positive control, showed a reduced HDV RNA level to 16–25% compared to the WT (Fig 2B, L31A). Among the variants, 16 alanine mutants showed the infection signal below 50% of the control, with four of them showing less than 20% (Fig 2B, light green for <20% and green for 20–50%). The low viral receptor activity for the representative NTCP mutants (L31A, Y146A, G158A, Q264A, and F274A) was confirmed in HBV infection assay that detected intracellular HBV core antigen at 13 days post-inoculation by immunofluorescence analysis (Fig 2C). Among them, Y146 and G158 had already been reported as the amino acids involved in NTCP activity HBV infection [12,23–25,27] (Fig 1A, 1B), indicating the validity of our assay. Fig 2D plots these 16 amino acids (by green circles) in the TM structure diagram: They are located both on the bile acid tunnel TMs and the extracellular loops (Fig 2D).

### Capacity of 54 NTCP mutants for preS1 binding

**Fig 3:**
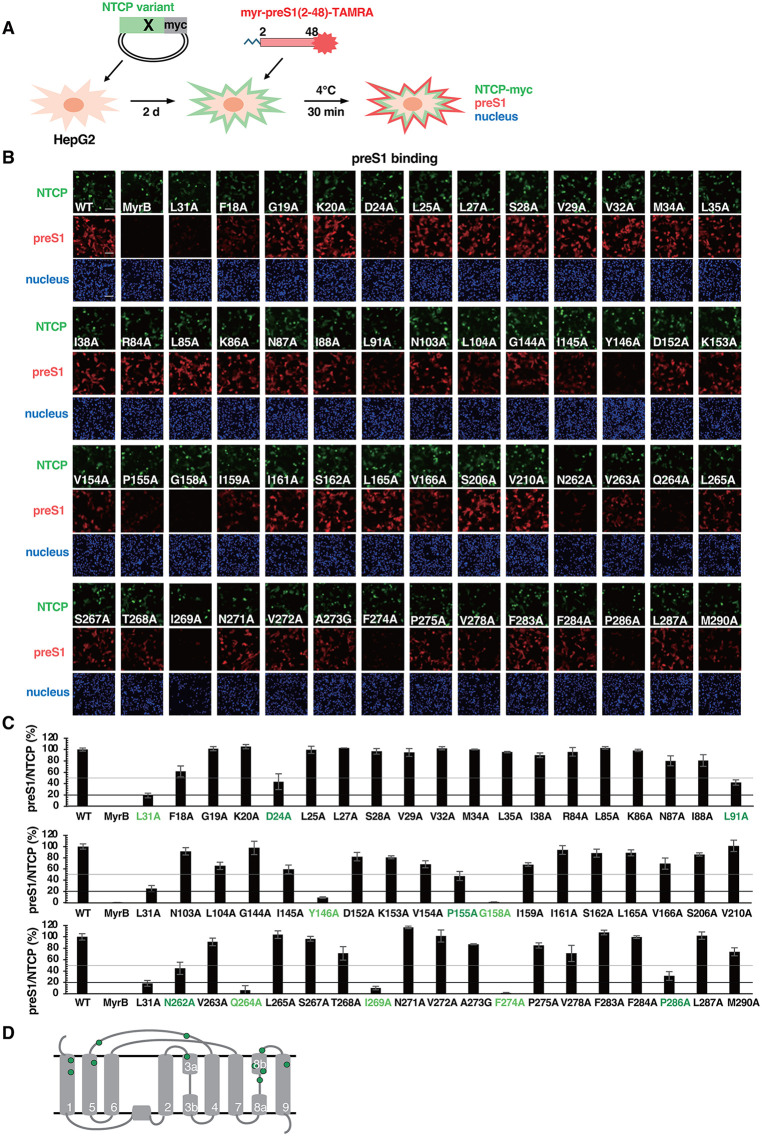
Capacity of 54 NTCP mutants for preS1 binding. **(A)** Schematic representation of the preS1 binding assay in NTCP mutant-overexpressing cells. HepG2 cells overexpressing WT or the 54 NTCP mutants were incubated with TAMRA fluorescence-conjugated myristoylated preS1(2-48)-peptide (preS1-TAMRA) for 30 min at 4˚C to allow preS1-NTCP binding. TAMRA fluorescence as well as myc-tagged NTCP (NTCP-myc) and the nucleus were detected with anti-myc antibody and DAPI by immunofluorescence analysis. **(B, C)** Fluorescence images of cell-bound preS1 (red), NTCP-myc (green) and the nucleus (blue) are shown in (B). Scale bar, 100 μm. Quantification of the relative red fluorescence area divided by green fluorescence area for each NTCP is indicated in (C). Gray and black lines indicate the 50% and 20% of the WT signal, respectively. Mutants showing the 20-50% and <20% signal are shown in green and light green, respectively. **(D)** Positions of 11 amino acids showing the signals less than 50% of the control by alanine mutation are shown in green in the TM structure diagram.

To examine the preS1 binding activity of NTCP mutants, we transfected HepG2 cells with the expression plasmid for myc-tagged WT or the alanine mutant NTCPs and then treated with N-terminally myristoylated preS1(2–48) labelled with TAMRA (preS1-TAMRA) at 4°C for 30 min. The cells were then fixed, permeabilized, and probed with anti-myc (NTCP, green), DAPI (nucleus, blue) as well as TAMRA fluorescence (preS1, red) by immunofluorescence analysis (Fig 3A and 3B). In this assay, the preS1 signals (red) for WT were overlapped with the NTCP signals (green), indicating that preS1 binding depends on NTCP WT expression, and the preS1 signals completely disappeared upon Myrcludex-B treatment, as a control experiment (Figs 3B, and enlarged image in S1). From the data, we calculated the preS1 binding rates with red florescence area (preS1) divided by green florescence area (NTCP), and the relative preS1 binding rates to that for WT are shown in Fig 3C. As a positive control, the L31A NTCP mutant [19], displayed a lower preS1 binding with 18–26% signals of WT (Fig 3B and 3C). In this condition, 11 mutants including L31A showed a reduction in preS1 binding rates below 50%, among which six reduced below 20% of WT (Fig 3B and 3C, light green for <20% and green for 20–50%). The Y146A and G158A mutants also showed a preS1 binding activity of less than 20% of WT, as reported [12,23–25,27]. These 11 amino acids were plotted in the TM structure diagram (Fig 3D, green circles), which are located both on the bile acid tunnel TMs and the extracellular loops. All of them are reasonably included in the amino acids important for supporting HDV infection in Fig 2.

### Capacity of 54 NTCP mutants for bile acid uptake

**Fig 4:**
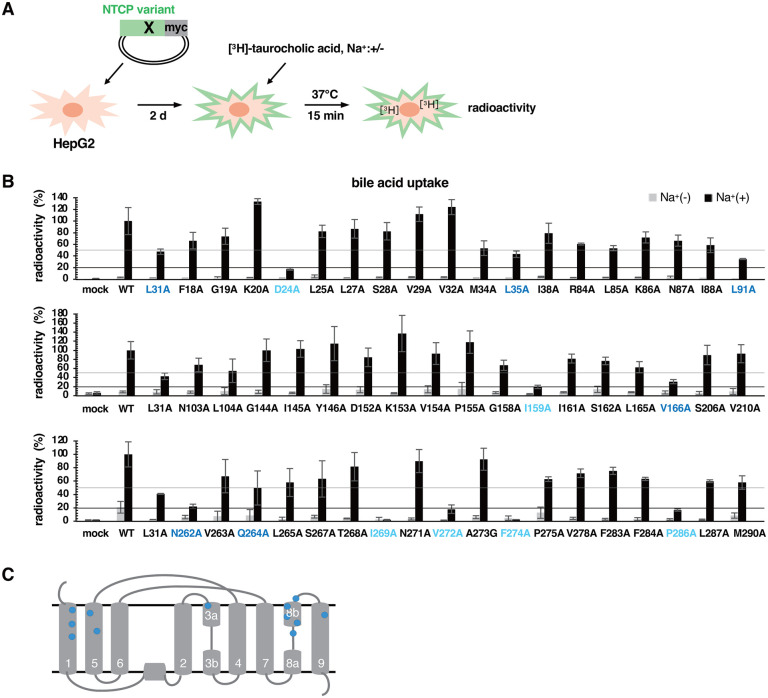
Capacity of 54 mutants for bile acid uptake. **(A)** HepG2 cells overexpressing WT or the 54 NTCP mutants were incubated with [^3^H]-taurocholic acid either in the presence or absence of sodium for 15 min at 37˚C to measure the intracellular radioactivity. **(B)** Intracellular radioactivity is shown by setting that in cells overexpressing WT in the presence of sodium as 100%. The gray and black bars indicate the values in the absence and presence of sodium, respectively. The sodium-dependent bile acid uptake indicates the NTCP activity. Gray and black lines indicate 50% and 20% of the WT signal, respectively. Mutants showing the 20-50% and <20% signal are shown in blue and light blue, respectively. **(C)** Positions of 12 amino acids showing the signals less than 50% of the control by alanine mutation are shown in blue in the TM structure diagram.

We examined the bile acid uptake activity by incubating HepG2 cells overexpressing WT or the mutant NTCPs with [^3^H]-taurocholic acid (TCA) in the presence or absence of sodium at 37°C for 15 min, followed by detection of intracellular radioactivity (Fig 4A). As bile acid uptake in the presence of sodium indicates the transporter activity of NTCP. The radioactivity in the cells overexpressing WT in the presence of sodium was designated as 100% in the graph (Fig 4B, black), and radioactivity level in the absence of sodium as background (Fig 4B, gray). In this assay, the N262A mutant, a position closely contacting with the bile acid pocket [24], showed an impaired activity at 22% of WT (Fig 4B, N262A), validating this bile acid uptake assay. Among the variants, 12 mutations reduced the bile acid uptake activity to below 50%, with six of them less than 20% compared with WT (Fig 4B, light blue for <20% and blue for 20–50%). The plotting on the TM structure diagram (Fig 4C) indicates that these 12 amino acids are predominantly located facing on the bile acid tunnel or its surrounding region, likely showing that the bile acid uptake activity does not require most extracellular loop regions of NTCP.

### Cell surface localization of 54 NTCP mutants

**Fig 5:**
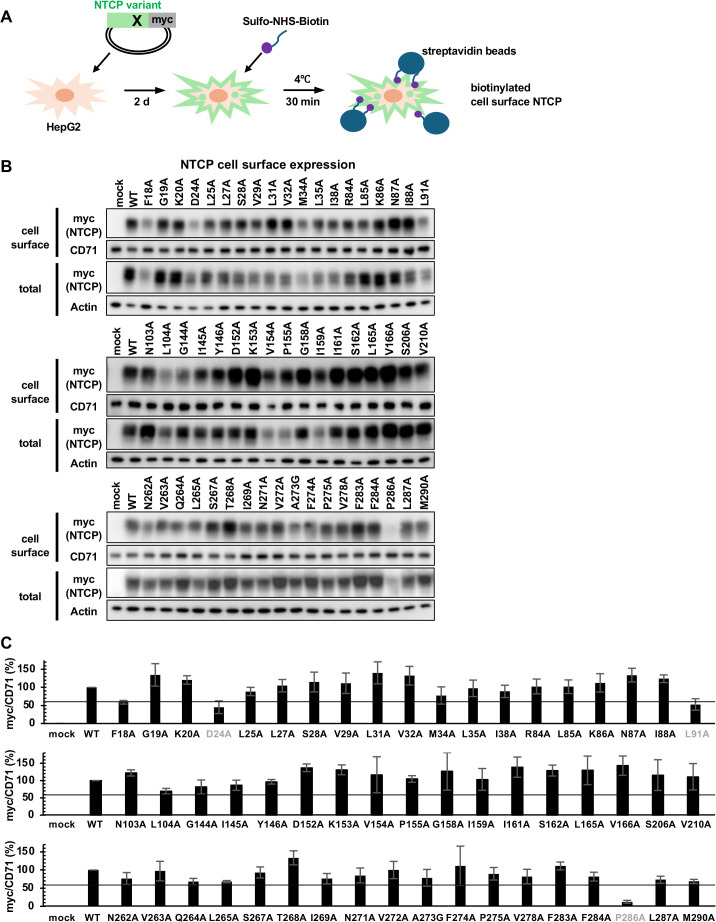
Cell surface protein expression of 54 mutants. **(A, B, C)** HepG2 cells overexpressing WT or the 54 NTCP mutants were incubated with the biotinylation buffer for 30 min at 4˚C to allow biotinylation of cell surface proteins, followed by washing, lysing cells, and pull down with streptavidin beads. Cell surface NTCP and CD71 as an internal control in the pull-down fraction were detected by immunoblotting using anti-myc and anti-CD71 antibodies (B, upper). In total cell lysate without biotinylation and pull down, NTCP and actin as an internal control were also detected using anti-myc and anti-actin antibodies (B, lower). In (C), band intensities of NTCP in the pull-down fraction quantified by densitometry were divided by that of CD71 in the pull-down fraction, and are shown in the graph as relative values by setting that in cells overexpressing WT as 100%. Black lines indicate 60% of the WT signal.

To evaluate whether alanine substitutions could affect the cell surface localization as well as NTCP expression or stability itself, we treated the transfected HepG2 cells with membrane impermeable-biotinylation reagent at 4°C for 30 min to allow selective biotinylation of cell surface proteins, followed by streptavidin-mediated pull down and immunoblotting to detect cell surface NTCP, and CD71 (transferrin receptor) as a cell surface internal control (Fig 5A). Relative cell surface NTCP expression was determined by normalizing the band intensity of NTCP by that of CD71 (Fig 5B, upper panels) as shown as a graph in Fig 5C. We also detected NTCP in total cell lysate without biotinylation as a reference by immunoblotting (Fig 5B, lower panels). The relative NTCP expression levels of the NTCP variants were variable, with most of them expressed at more than 60% of WT, except for F18A, D24A, L91A and P286A, showing 58%, 44%, 52% and 12% of WT (Fig 5B and 5C). It is not clear whether these lower expression levels are insufficient or still enough for presenting each activity, but F18A, which showed 58% of cell surface NTCP expression (Fig 5C) was still active for supporting HDV infection, preS1 binding, and bile acid uptake (Figs 2–4). In contrast, D24A, L91A and P286A showed impairment of all the activities (32%, 35% and 16% of HDV infection, 43%, 42% and 31% of preS1 binding, and 18%, 34% and 17% of bile acid uptake), possibly due to the reduced cell surface NTCP expression, rather than the altered functionality of NTCP protein. We then neglected D24A, L91A and P286A (Fig 5C, gray) from the interpretation of data.

### Mapping of the functional amino acids involved in viral infection, preS1 binding, and bile acid uptake

We used the same assays as shown in Figs 2–5 using Huh-7 cells to address the possible impact of cellular background on the NTCP activity profile. As shown in S2 Fig, L31A, Y146A, G158A, Q264A, I269A, and F274A, but not V32A and N271A, showed reduced activities for supporting HDV infection (2–23% compared with the WT) (S2A Fig) and preS1 binding (0–30%) (S2B Fig), with similar cell surface expression levels for all these NTCP mutants (S2D Fig). As well, mutations, L31A, Q264A, I269A, and F274A, but not Y146A, G158A, V32A, and N271A reduced the bile acid uptake activity to below 50% (S2C Fig). These results using Huh-7 cells were totally consistent with those using HepG2 cells, indicating that the activities of NTCP mutants are not reflected by cellular background.

**Fig 6:**
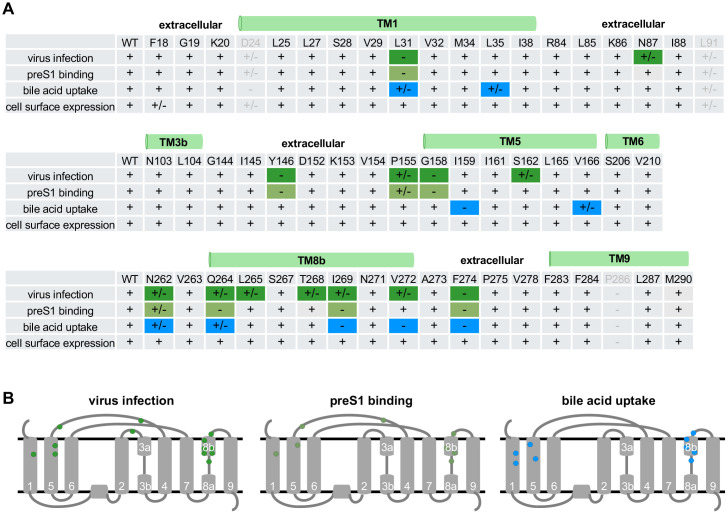
Profile of 54 NTCP mutants for capacity to support viral infection, preS1 binding, bile acid uptake, and cell surface expression. **(A, B)** Capacity of 54 NTCP mutants to support virus infection, preS1 binding, bile acid uptake, and cell surface expression is summarized based on the results shown in Figs 2–5. Capacities that were >50% of WT are shown as +, and those that were 20-50% and <20% are shown as +/- and -, respectively (A). +/- and - are highlighted by dark green, green, and blue for the capacity supporting virus infection, preS1 binding, and bile acid uptake, respectively. D24A, L91A, and P286A mutants are excluded (shown by gray) because of the low cell surface expression levels. Amino acid positions showing +/- and – by alanine mutation are shown in each TM structure diagram (B).

The activities of NTCP-mediated viral infection, preS1 binding, and bile acid uptake as well as cell surface expression of each alanine mutant and WT are summarized in Fig 6A and S1 Table. For suggesting the amino acids involved in each function, we set the criteria those reducing the function to below 50% by their alanine substitution, rather than the statistical significance that raises many amino acids showing minor and non-essential contributions. Except for D24, L91, and P286, of which alanine substitutions reduced cell surface expression, 13 amino acids appear to be instrumental for viral infection, among which L31, N87, Y146, G158, N262, Q264, T268, V272, and F274, already reported as potential sites associated with the viral receptor function, and P155, S162, L265, and I269 were identified as the novel sites (Fig 6A). The preS1 binding assay identified eight amino acids, all of which were contained as the sites supporting HDV infection. Nine amino acids were identified as involved in the bile acid transporter function (Fig 6A). Note that there may be additional amino acids required for the transporter function that were not examined in this study, since we focused on the NTCP amino acids closely contacting to the preS1 amino acids.

We plotted the above amino acids suggested to be involved in the three NTCP functions on the TM structure diagram in Fig 6B. The functional overlapping of the viral receptor function with the bile acid transporter function, as well as the viral receptor function without involvement in the preS1 binding, are discussed (see Discussion section).

### Reduction in the viral receptor function by SNP-associated NTCP variants

**Fig 7:**
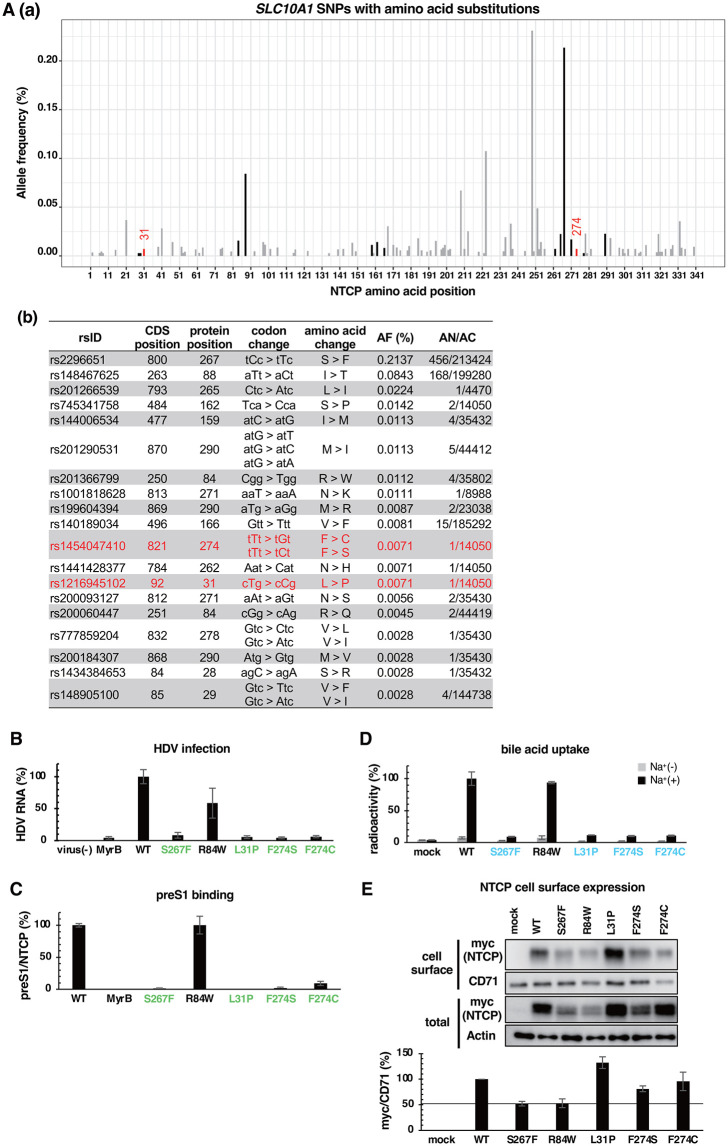
Reduced viral receptor function of SNP-associated NTCP variants. **(A)** Allele frequencies (AF) of 114 single nucleotide polymorphisms (SNPs) causing amino acid substitutions at NTCP positions are plotted on the vertical axis, with amino acid positions indicated on the horizontal axis. Colors represent preS1 binding activity based on alanine substitution results: red, black and gray indicate < 20%, ≥ 20% activity of the WT and sites not examined, respectively (a). Among the 114 extracted SNPs, 19 SNPs corresponding to the 54 NTCP sites examined in this study are shown. SNP identification numbers (rsIDs), CDS (Coding DNA Sequence)/protein positions, codon/amino acid changes, AF (%), and AC (allele count)/ AN (allele number) are listed in descending order of allele frequency (b). **(B–E)** HDV infection (B), preS1 binding (C), bile acid uptake (D), and NTCP cell surface expression (E) were examined for NTCP WT or that having a substitution L31P, F274S, F274C, R84W, and S267F as a positive control, as shown in Figs 2A, 3A, 4A, and 5A.

The *SLC10A1* gene has a lot of SNPs that cause substitution of an amino acid in NTCP and potentially affect NTCP functions. We extracted 114 SNPs located within the open reading frame of the *SLC10A1* gene that are associated with amino acid substitutions in NTCP from the NCBI ALFA (Allele Frequency Aggregator) database. Fig 7Aa shows these SNPs along with their allele frequencies (AF) at each SNP position. Among these, 19 SNPs cause amino acid substitution at the above 54 NTCP sites examined in this study (Fig 7A). Since alanine substitution at L31 and F274 (among the 19 sites) drastically reduced preS1 binding and viral infection below 20%, we focused on the two SNPs, rs1454047410 and rs1216945102, which cause F274C/S and L31P substitutions, respectively (Fig 7A). We also used a SNP-associated R84W as a reference –an alanine substitution at this site unaffecting preS1 binding and HDV infection (Figs 2 and 3)– as well as S267F as a positive control for the functionally deficient NTCP variant [14]. To examine the impact of these SNP-associated amino acid substitutions on viral receptor function, we prepared the expression plasmid encoding these NTCP variants and overexpressed them in HepG2 cells to assess NTCP activities using the same assays as shown in Figs 2–5. As shown in Fig 7B–7D, L31P and F274C/S showed remarkable reduction in susceptibility to HDV infection, preS1 binding, and bile acid uptake, as was the case with S267F (Fig 7B–7D). As the relative cell surface expression levels of L31P and F274C/S were above 80% of that for the WT (Fig 7E), the two NTCP variants, F274C/S and L31P, caused by *SLC10A1* SNPs, rs1454047410 and rs1216945102, respectively, are less active or deficient for supporting preS1 binding and bile acid transporter function.

### Molecular dynamics simulation explaining the impact of substitutions at position 274 for preS1-NTCP binding

**Fig 8:**
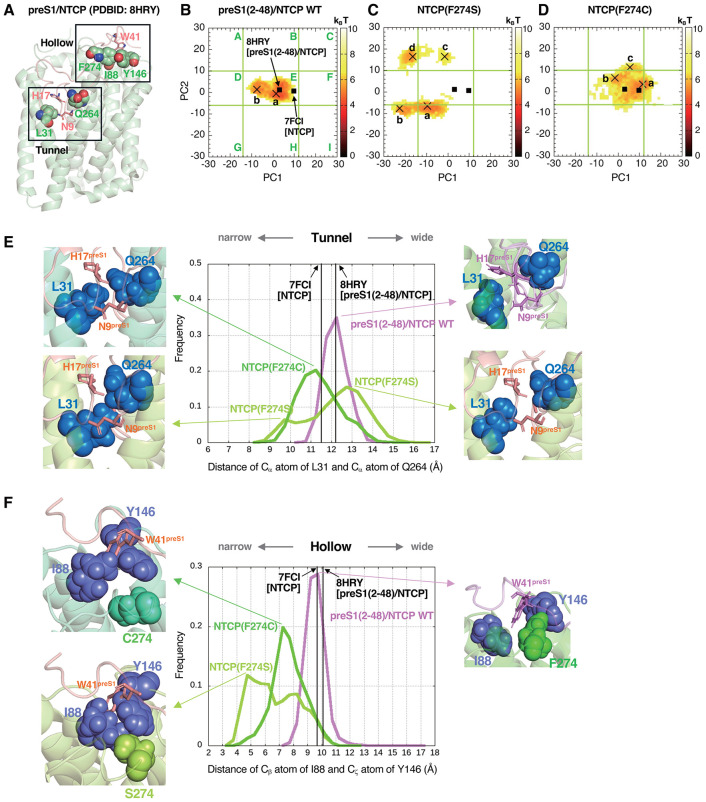
Impact of the SNP-associated amino acid substitutions at position 274 of NTCP on preS1 binding in molecular dynamics simulations. **(A)** Cryo-EM structure of the preS1(2-48)/NTCP WT complex. NTCP is shown by pale green cartoons, with its residues L31, Q264 (tunnel region) and I88, Y146, and F274 (hollow region) shown as spheres. The preS1 residues N9, H17 in the tunnel, and W41 in the hollow are shown by pink sticks. **(B–D)** PCA showing the structural distribution of NTCP for preS1(2-48)/NTCP WT (B), its F274S (C) and F274C (D) variants during MD simulations. Frequencies are shown by color (brown to white; high to low). The cryo-EM structures are shown by black squares [NTCP (PDB: 7FCI)) and preS1(2-48)/NTCP (PDB: 8HRY)], and the minima in basins are shown by black X mark. Nine areas (A to I) are defined so that the structural distribution for preS1(2-48)/NTCP WT is surrounded by both PC1 and PC2. **(E)** Width of the bile acid tunnel. Frequency of the distance of C_α_ atoms between L31 (TM1) and Q264 (TM8b) for preS1(2-48)/NTCP WT (violet), NTCP(F274S) (light green), and NTCP(F274C) (bright green) during MD simulations are shown. The snapshot structures at the peaks are illustrated, with superimposing preS1 structure in the cryo-EM preS1(2-48)/NTCP WT structure as an eye guide (pink) to the apo snapshots of NTCP (F274S or F274C). **(F)** Width of the outer-surface hollow. Frequency of the distance between the C_β_ atom of I88 and the C_ζ_ atom of Y146 for preS1(2-48)/NTCP WT (violet), NTCP(F274S) (light green), and NTCP(F274C) (bright green) during MD simulations. The snapshot structures at the peaks are illustrated, with superimposing preS1 structure to the snapshot for NTCP (F274S or F274C) as shown in (E). The residue of W41^preS1^ is represented as pink stick. The superimposition between the snapshot of apo NTCP and the cryo-EM structure is done using the PyMOL alignment command.

We have already reported that L31 faces the bile acid tunnel surface in TM1 and plays a significant role in preS1 binding [18]. To get an insight into how the SNP-associated NTCP variant at position 274 could affect the preS1 binding activity, we performed molecular dynamics (MD) simulations using substitutions at position 274 to either cysteine or serine as well as the WT using the NTCP models embedded in a membrane-water system (S3A Fig). We analyzed the impact of the mutations on the overall NTCP structure (S1A Fig) in the apo state, because of almost the complete loss of preS1 binding capacity by F274 substitutions. The below analyses especially focused on 1) the extracellular “hollow” that is formed by I88, Y146, and F274, where W41 of preS1 (W41^preS1^) binds (Figs 8A and S1B) and 2) the bile acid tunnel area between L31 at TM1 and Q264 at TM8, where 7–19 aa of preS1 is inserted (Figs 8A and S1B, S1C).

Structural distributions of NTCP during the MD simulations were changed by the F274C or F274S substitution in comparison with the preS1(2–48)-bound NTCP WT (Fig 8B–8D): In the principal component analysis (PCA) for the NTCP structure distributions, nine areas denoted as A to I were defined based on the surrounding area of the NTCP distributions appeared for preS1(2–48)/NTCP WT (Fig 8B), i.e., the area E covers all the structures of NTCP WT during the simulations, close to the cryo-EM preS1-binding structure (black square). In contrast to this pattern, NTCP(F274S) structure distribution appeared different, with more diverse and almost no distribution in the area E, suggesting the NTCP(F274S) structures far from the preS1-bound NTCP states (Fig 8C). F274 closely contacts with Y146 and I88 and forms the outer-surface hollow on the extracellular NTCP (the distance between I88 C_β_ and Y146 C_ζ_ is 9–10 Å) that fits W41^preS1^ (Figs 8A, 8F, S1B and S1D), however, F274S mutant constitutes narrower hollow (the distance between I88 C_β_ and Y146 C_ζ_ is 4–6 Å) that was too narrow to permit W41^preS1^ binding (Fig 8F). In addition, F274S substitution affected the structure of bile acid tunnel: NTCP(F274S) temporarily showed a narrower or wider bile acid tunnel than NTCP WT, as shown by the distance between L31 in TM1 and Q264 in TM8b (Fig 8E). Typical snapshots corresponding to the four basins on the PC surface denoted as a-d were shown in S2B Fig, and these results explained the low preS1 binding activity.

NTCP(F274C) distributed at the area E, but also at the area B, F, and H with more diverse structure than WT (Fig 8D), and its structural features of both the extracellular hollow and the bile acid tunnel were changed: The hollow width was narrower by ~2 Å than that for WT (Fig 8F). In addition, the bile acid tunnel was also slightly narrower (Fig 8E). The typical snapshots corresponding to three points on the PC surface, denoted as a-c, were shown in S2C Fig, of which structural features were disadvantageous for preS1 binding.

These computational analyses suggest that the SNP-associated substitutions at position 274 prevent the maintenance of the structural features necessary for stable binding of preS1 in both the outer-surface hollow and the bile acid tunnel, potentially leading to the drastic decrease in the NTCP receptor function.

## Discussion

**Fig 9:**
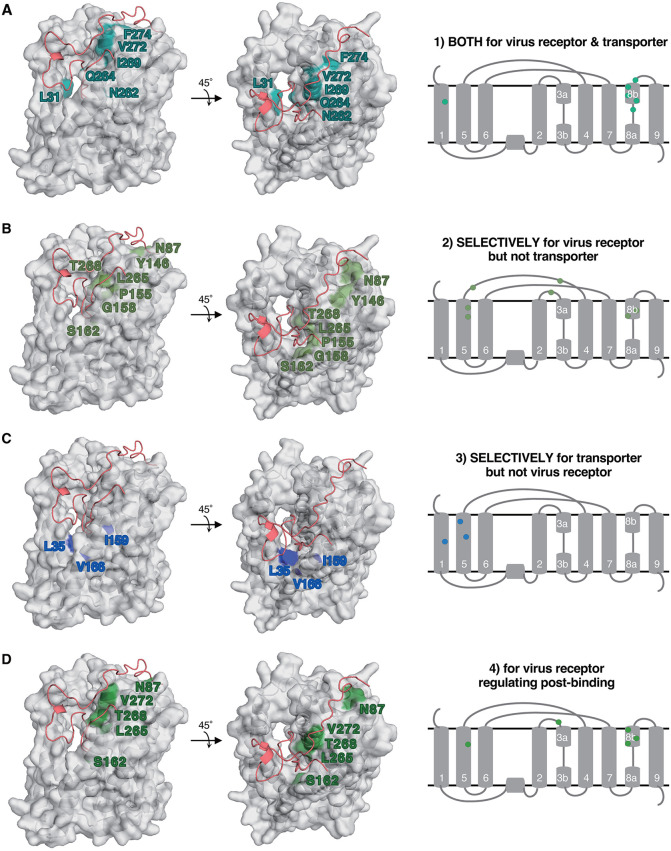
Overlapping functionality of NTCP as a viral receptor and bile acid transporter. **(A)** Six amino acids functioning for both supporting viral infection and bile acid uptake are shown by turquoise, located at TM1 and 8b facing the bile acid tunnel. **(B)** Seven amino acids specifically involved in supporting viral infection but not bile acid uptake are shown by light green, located on the extracellular loops and at TM5 and 8b. **(C)** Three amino acids specifically involved in bile acid uptake but not viral infection are shown by blue, located at TM1 and 5. **(D)** Five amino acids important for supporting viral infection but not preS1 binding are shown by green.

In this study, we focused on 54 amino acids in NTCP that closely contact to HBV preS1 peptide within 4.5 Å and found them widely located at TM1, 3, 5, 6, 8, and 9 facing on the bile acid tunnel and at loops TM2–3, TM4–5, and TM8–9 on the extracellular surface. Among them, however, amino acids that were suggested to be functionally involved in HDV infection are specifically located at TM1, 5, 8, and the extracellular loops, whereas those suggested to function for bile acid uptake are located at TM1, 5, 8, and their peripheral areas (Fig 9A and 9B). The amino acids involved in the two functions are not identical, but partially overlapped. Consistent with the previous report suggesting that several amino acids responsible for bile acid uptake were also active for preS1 binding [14], our results further provides a more comprehensive functional map of the NTCP protein that explains the role of amino acids with structural visualization (Fig 9). Interestingly, all amino acids supporting both infection and bile acid uptake are exclusively located at TM8 and its peripheral area (N262, Q264, I269, V272, and F274) in addition to L31 in TM1 (Fig 9A). In contrast, those specifically involved in susceptibility to infection but not bile acid uptake are located at the extracellular loops distal to the bile acid tunnel (N87 and Y146), as well as at TM5 (P155, G158, and S162) and TM8 (L265 and T268) (Fig 9B). And those specifically involved in the bile acid uptake among the focused amino acids are only within TM1 (L35) and TM5 (I159 and V166), but not at TM8 (Fig 9C). In summary, among the bile acid tunnel inner-surface including TM1, 5, and 6 in the panel domain and TM3, 8, and 9 in the core domain, we propose that: 1) TM1, 5, and 8, and the extracellular surface contain amino acids responsible for supporting virus infection and bile acid uptake, 2) TM8 contains major amino acids that are overlapping and involved in both functions, 3) TM5 does not have any amino acids responsible for both functions but those specifically for one function, 4) in TM1, only L31 is involved in both functions, and 5) the extracellular surface contains an amino acid island at the distal area to the bile acid tunnel (Fig 9B, N87, Y146) that specifically functions for susceptibility to infection. Thus, the functional region for both the virus receptor function and the bile acid transporter function involves TM1 and 8, but not at TM5 and the extracellular loops.

How do the amino acids identified in this study regulate HBV infection? Among the 13 NTCP amino acids that are potentially involved in HBV/HDV infection, eight regulate preS1 binding and five do not. This suggests that NTCP is instrumental for preS1 binding but also other steps, likely the step after preS1 binding up to membrane fusion. The latter five amino acids, not involved in preS1 binding, face either the cavity between TM5 and 8 (S162, L265, T268, and V272) or the extracellular side at the TM2–3 loops (N87) (Fig 9D). We have so far reported that the post-preS1 binding process including internalization and membrane trafficking for endosomal sorting depends on an NTCP-interacting cofactor, epidermal growth factor receptor, an NTCP-interacting cofactor [29,30]. The subsequent membrane fusion was recently reported to involve host ERp57, although it is unknown whether it interacts with NTCP [31]. The deficient NTCP mutants identified here may reduce the interaction with these entry cofactors and the activity to support a post preS1-binding step, however, these five amino acids face the bile acid tunnel or the extracellular side, which are unlikely to affect an interaction with NTCP partner(s). A previous study that examined the effect of swapping amino acids between human and monkey NTCP speculated that the 84–87 aa motif regulated the internalization step, although it has not been experimentally demonstrated [25], which is consistent with the above results showing the possible role of N87 at the post-binding step. In future study, these NTCP mutants would be useful as a tool to analyze the regulation mechanism of the post preS1-binding step during the virus entry.

Based on the SNPs of the *SLC10A1* gene registered in the public database, we identified SNPs-associated NTCP rare variants with impaired virus receptor activity, F274C/S and L31P, which are produced by the rare *SLC10A1* SNPs, rs1454047410 and rs1216945102, respectively. Although the transporter activity of more than 30 NTCP variants produced by *SLC10A1* SNPs has been so far reported [32], the virus receptor function has not been analyzed, except for the S267F mutation associated with a low preS1 binding activity [14]. Functional impairment of S267F is speculated to be due to the induction of steric hindrance by the bulky phenylalanine at position 267 facing on the narrow bile acid tunnel that is important for preS1 embedding. In contrast, we suggest by MD simulation that the replacement of phenylalanine at position 274 by less-bulky cysteine or serine rendered the NTCP structure unsuitable for preS1 binding. The substitutions at position 274 particularly 1) impaired the stable formation of the outer-surface hollow where W41^preS1^ fits, and 2) changed the width of the bile acid tunnel into which the 7–19 aa region of preS1 tightly inserted. Thus, efficient preS1 binding and the resulting virus infection require both the preS1’s insertion into the narrow bile acid tunnel and a stable interaction onto the extracellular outer-surface of NTCP. The diverse NTCP compatibility to preS1 by *SLC10A1* SNPs may affect HBV/HDV infection susceptibility and pathogenesis of the related liver diseases, as the case with rs2296651 (causing S267F substitution), which is a future subject to be analyzed.

In this study, we mapped the NTCP amino acids involved in the HBV/HDV receptor function and the bile acid transport. Limitation of this study includes the supplement of PEG8000 in our infection assay, which facilitates virus-cell interaction to enhance infection efficiency [33], but may cause some artificial effect, even though PEG8000 is widely used in the standard HBV/HDV infection assays [34]. Also, alanine mutation analysis is not necessarily able to identify all the *SCL10A1* SNP-associated substitutions that affect the activity of viral receptor and bile acid transporter functions, since such activities can depend on the replaced amino acid types and their size, as the case with the mutation at S276, of which substitution to F but not A impairs the activities as a viral receptor and bile acid transporter. This analysis reveals the overlapping pattern of the dual function of NTCP and suggests the possible impact of *SLC10A1* SNPs on virus infection. These evidences provide the basic information useful for understanding the dual functionality of NTCP and for developing selective antiviral drugs that separate the antiviral activity from the bile acid uptake function.

## Materials and methods

### Selection of NTCP amino acids contacting with preS1

Based on the preS1(2–48)/NTCP structure (PDB: 8HRY) [21], we selected the amino acids in NTCP that are located within 4.5 Å from any amino acids of preS1(2–48) by PyMOL software. The closest interatomic distances between the selected NTCP amino acids and the counterpart preS1 amino acids were measured and are shown in Table 1. The 4.5 Å threshold was chosen to include direct contacts and near contacts that plausibly contribute to binding or gating while accommodating side-chain mobility and coordinate uncertainty. Sensitivity analyses with 4.0 Å and 5.0 Å produced qualitatively similar results.

### Cell culture

HepG2 cells were cultured in Dulbecco’s modified Eagle’s medium (D-MEM)/F-12, GlutaMAX (Thermo Fisher Scientific) containing 10% fetal bovine serum (FBS), 100 U/mL penicillin–streptomycin,10 mM HEPES, and 5 μg/mL insulin at 37°C in 5% CO_2_. Huh-7 cells were cultured in Dulbecco’s modified Eagle’s medium (DMEM; Invitrogen) supplemented with 10% FBS, 100 U/mL penicillin–streptomycin, 0.1 mM nonessential amino acids (Invitrogen), 1 mM sodium pyruvate, and 10 mM HEPES (pH 7.4) at 37°C in 5% CO_2_.

### Plasmid construction

The expression plasmid for the wild type NTCP was described previously [23]. The plasmids carrying a point mutation to alanine were constructed by oligonucleotide-directed mutagenesis using PrimeSTAR Mutagenesis Basal Kit (TaKaRa), according to the manufacturer’s protocol and inserted between the KpnI and XbaI sites of the pEF4-myc-His vector. All the prepared plasmids were sequenced for confirmation.

### NTCP expression in the cells

HepG2 cells transiently expressing the wild type or the mutant NTCP were generated by transfection with the NTCP expression plasmids using ViaFect Transfection Reagent (Promega) according to the manufacturer’s protocol in the medium without penicillin-streptomycin [24]. Huh-7 cells transiently expressing the wild type or the mutant NTCP were generated by transfection with the NTCP expression plasmids using TransIT LT1 Reagent (Mirus Bio) according to the manufacturer’s protocol. Assays were started at two days post-transfection.

### Biotinylation of cell surface proteins

The cells were treated with the biotinylation reagent, EZ-Link Sulfo-NHS-LC-Biotin (Thermo Fisher Scientific) at 4°C for 30 min to allow the biotin labelling of proteins on the cell surface, but not inside the cells [24]. After removing the reagent, PBS with 0.1% bovine serum albumin (BSA) was added to quench the reaction. The cells were lysed with the buffer [150 mM NaCl, 50 mM Tris-HCl (pH 7.4), 5 mM EDTA, 1% NP-40, protease inhibitor cocktail (Roche), 0.1% SDS] at 4°C for 20–30 min. After centrifugation, the cell lysates were added with the BSA-blocked streptavidin beads at 4°C for 2 h, followed by washing and elution with 2 mM biotin. To examine the protein production levels in total cell simultaneously, total fraction without streptavidin pull-down was also recovered by directly lysing cells without treating with the above biotinylation reagent.

### Immunoblot analysis

For detecting NTCP, samples were digested with peptide-N-glycosidase F (New England Biolabs) 6.25 U/μL to remove glycosylation [24]. Immunoblot analysis was conducted by using 3,000-fold diluted anti-myc [c-myc (9E10), Santa Cruz Biotechnology], 25,000-fold diluted anti-actin (sigma), and 10,000-fold diluted anti-CD71 (abcam) as primary antibodies, and anti-mouse IgG-HRP (Thermo Fisher Scientific), anti-rabbit IgG-HRP (Thermo Fisher Scientific) as secondary antibodies to visualize proteins with WSE-6300 LuminoGraph III (ATTO).

### PreS1 binding assay

The cells were incubated with C-terminally TAMRA-conjugated and N-terminally myristoylated-preS1 peptides consisting of 2–48 aa (preS1-TAMRA) at 10 nM for 30 min at 4°C to allow binding to the cell surface but not internalization [24]. Myrcludex-B (Selleck Biotech), an HBV-cell attachment inhibitor, was used as a positive control to block preS1-mediated specific cell binding. After fixation with 4% paraformaldehyde and permeabilization with 0.01% digitonin (Wako), the cells were treated with 0.02% 4′,6-diami-dino-2-phenylindole (DAPI) and 3,000-fold diluted Alexa Fluor 488 Anti-myc antibody (abcam) to detect preS1-TAMRA, the nuclei, and myc-tagged NTCP with fluorescence microscopy (BZ-X810, Keyence). For quantification of preS1 binding activity of each NTCP variant, the fluorescence areas of NTCP and preS1 were quantified with BZ-X810 analyzer (Keyence), the NTCP signal was isolated from the merged images and quantified, followed by quantification of the preS1 signal area within the isolated NTCP signal area. The preS1 area was divided by the NTCP area. The relative values to that for the WT expressing control cells were calculated with each value divided by that for the WT to show the percentage to the WT as a graph (Fig 3C).

### HDV infection assay

NTCP-dependent viral entry was evaluated by the HDV infection assay as described previously [23]: HDV (genotype I) used as an inoculum was prepared by recovering the culture supernatant of Huh-7 cells transfected with the plasmids for an HDV genome and for HBV surface antigen (The HDV expression plasmid was kindly provided by Dr. John Taylor at Fox Chase Cancer Center).

HepG2 cells overexpressing the wild type or the mutant NTCP were inoculated with HDV with 5% polyethylene glycol-8000 for 18 h, followed by washing and culturing for 6 days to detect intracellular HDV RNA. Myrcludex-B was used as a positive control to inhibit viral entry that was co-treated during the HDV inoculation. During the culture, the medium was replaced every three days.

### HBV infection assay

For this assay, HBV (genotype D) derived from the culture supernatant of Hep38.7-Tet cells prepared as described [35], was used as the inoculum. HepG2 cells overexpressing the wild type or the mutant NTCP were inoculated with HBV using 4% polyethylene glycol-8000 under treatment with or without MyrB. After 18 h, free viruses were washed out, and the cells were cultured for 12 days. During the infection assay, culture medium was replaced every three days. After fixation with 4% paraformaldehyde and permeabilization with 0.3% Triton X-100, the cells were treated with 0.02% DAPI and anti-HBc antibody to detect the nuclei, HBV core antigen (HBc) with fluorescence microscopy (BZ-X810, Keyence).

### RNA extraction and real time RT-PCR

RNA was extracted using RNeasy Mini Kit (250) (QIAGEN) according to the manufacturer’s protocol. HDV RNA was quantified by real time RT-PCR using THUNDERBIRD Probe One-step qRT-PCR Kit (TOYOBO) and 5′-GGACCCCTTCAGCGAACA-3′ and 5′-CCTAGCATCTCCTCC TATCGCTAT-3′ as a primer set and 5′- AGGCGCTTCGAGCGGTAGGAGTAAGA-3′ as a probe with QuantStudio 3 Real-Time PCR System (Thermo Fisher Scientific). To quantify the activity of each NTCP for supporting HDV infection, intracellular HDV RNA values for each NTCP relative to that for WT are calculated and are shown (Fig 2B).

### Transporter assay

The cells were incubated with 0.1 µM [^3^H]-taurocholic acid in the buffer (4.8 mM KCl, 1.2 mM KH_2_PO_4_, 1.2 mM MgSO_4_, 1.3 mM CaCl_2_, 2.6 mM D-glucose, and 25 mM HEPES) either in the presence or absence of 125 mM Na^+^ at 37°C for 15 min. After washing out, the cells were lysed with 0.05% SDS and then added 1 mL of Insta-Gel Plus (Revviy) to measure intracellular radioactivity using a liquid scintillation counter (Perkin Elmer).

### Molecular dynamics simulation

We performed four systems: preS1(2–48)/NTCP WT complex, apo NTCP(WT), apo NTCP(F274S), and apo NTCP(F274C). The initial structure of the MD simulations was prepared using the cryo-EM structure of preS1(2–48)/NTCP WT complex (PDB: 8HRY). In the apo state, the initial structure of NTCP was prepared by cutting the preS1(2–48) moiety from the complex (PDB: 8HRY). The membrane-water systems were prepared using Membrane Builder module implemented in CHARMM-GUI [36–38]. During setup in CHARMM-GUI, missing hydrogen atoms were added, according to the protonation states of residues determined by their pKa values at preS1(2–48)/NTCP WT complex estimated by PROPKA implemented in PDB2PQR [39]: E257^NTCP^ was set to a protonated glutamic acid, and D16^preS1^ was set to a protonated aspartic acid. All histidine residues in the preS1/NTCP complex were set to neutral histidine, and all the histidine residues except for H57^NTCP^ and H17^preS1^ were protonated only N_δ_ atom (HSD in the charm force field), and H57^NTCP^ and H17^preS1^ were protonated only N_Ɛ_ atom (HSE in the charm force field). The N- and C-termini in NTCP were set to NH_3_^+^ and COO^–^, respectively. In apo NTCP, the same protonation state for preS1/NTCP complex was employed. In preS1, the myristoylation of the N-terminal (GLYM in the charm forcefield) was added and the C-terminal was set to COO^–^. F274C or F274S substitutions in NTCP were performed in the setup process. The complex or apo NTCP was embedded in a 70 Å x 70 Å 1-palmitoyl-2-oleoyl-glycero-3 -phosphocholin (POPC) bilayer in the x-y plane in the center of the rectangular unit cell. The orientation of NTCP relative to the membrane was set the same as that of NTCP deposited in the Orientations of Proteins in Membranes database (PDB: 7FCI). After filling the unit cell with water molecules (TIP3water model), sodium and chloride counterions (NaCl) were added with 150 mM ion concentration. The constructed system for the preS1/NTCP complex embedded in a membrane-water system is illustrated in S3A Fig and details of the system setup were summarized in S1 Table.

All-atom conventional MD simulations were performed and analyzed by using the MD program package GROMACS ver. 2021.4 [40] under periodic boundary conditions. The CHARMM36m force field [41–43], which is often used for membrane-protein systems, was used. Energy minimization and equilibration runs were performed according to the default setting in Membrane Builder; detailed conditions were the same as those of our previous simulations [44]. Then, three independent 1-μs production runs with different initial velocities and initial configuration were performed; each independent MD was performed from the energy minimization process. The NPT ensemble was used, and temperature and pressure were set at 300 K and 1 atm, respectively. The thermostat and barostat used were the Nose-Hoover scheme and the Parrinello–Rahman approach, respectively. The type of pressure coupling was semi-isotropic. The particle mesh Ewald method was used for evaluating electrostatic interactions, and the van der Waals interactions were smoothly truncated using the switching function within 10–12 Å range. Bond length involving hydrogen atoms was constrained by the P-LINCS algorithm, and a 2-fs timestep was used. MD simulations for the three systems were performed using the same procedure and conditions as described above. Snapshots were saved every 1 ns from a 1-μs MD simulation and used for trajectory analyses. Principal component analysis (PCA) of the NTCP structures was performed using C_α_ atoms for the 22–310 residues of NTCP after a structural alignment of the snapshots using the C_α_ atoms of 22–310 residues, and the PC axes were determined using three systems of apo NTCP MD simulations and four cryo-EM structures, i.e., three MDs for apo NTCP(WT), apo NTCP(F274S), and, apo NTCP(F274C), and four cryo-EM structures (PDB: 7FCI, 8HRY, 7ZYI, and 7PQG). Contributions of PC1 and PC2 against the total degree of freedom were 21.5% and 10.6%, respectively. Snapshots of preS1(2–48)/NTCP WT were projected onto the PC axes. The free energy landscapes shown as color maps (Fig 8B–8D) were evaluated by –ln*P*, where *P* is the existence probability calculated on the normalized histogram of 1 Å x 1 Å pixels on the PC1-PC2 surface. The representative structure of the basin “a” on the FEL was selected by the central structure of the cluster with the most members after clustering the structures at 1 pixel corresponding to the minimum of the basin, in which the hierarchical clustering of C_α_ and C_β_ atoms was performed, using the MMTSB tool [45]. In other basins, positions near local minima on the PC surface were selected, and the representative structure was chosen as was so for the basin “a”.

### Extraction of the *SLC10A1* SNPs

Allele frequencies for single nucleotide polymorphisms (SNPs) in the *SLC10A1* were obtained from the NCBI ALFA (Allele Frequency Aggregator) database (https://www.ncbi.nlm.nih.gov/snp/docs/gsr/alfa/). Using the VCF dataset (release 2, version 20201027095038), we extracted SNPs corresponding to the genomic region of *SLC10A1* (chromosome 14: 69775416-69797241) and allele frequencies (AF) for the total population (SAMN10492705) using BCFtools/view version 1.18. For each SNP, annotation data, including amino acid mutation patten, was obtained using the Ensembl Variant Effect Predictor (VEP v114.0, GRCh37) [46]. Only SNPs annotated as missense variants were retained for further analysis. Data manipulation was conducted using VariantAnnotation version 1.48.1 [47] in R 4.3.2 (R Foundation for Statistical Computing, Vienna, Austria).

## Supporting information

S1 Fig PreS1 binding capacity of NTCP mutants. The enlarged immunofluorescence images of Fig 3B are shown.
(PDF)

S2 Fig Activity of NTCP mutants for supporting viral infection, preS1 binding, bile acid uptake, and cell surface expression in Huh-7 cells. HDV infection (A), preS1 binding (B), bile acid uptake (C), and protein expression on cell surface and total fraction (D) were examined using Huh-7 cells overexpressing NTCP WT or its mutants, as described in Figs 2–5.
(PDF)

S3 Fig Structures of outer-surface hollow and the bile acid tunnel regions. (A) A membrane-water system for apo NTCP used in the MD simulations. NTCP is represented as green cartoon, membrane molecules are gray lines, and water, sodium, and chloride molecules are cyan, yellow, and green color spheres, respectively. (B) The outer-surface hollow formed by I88, Y146, and F274 is shown based on the cryo-EM structure of the preS1(2–48)/NTCP WT complex. W41 in preS1 is docked in this extracellular hollow. Green and pink cartoon indicate NTCP and preS1, respectively. (C) The tunnel region formed by TM1 (L31 as a marker) and TM8b (Q264 as a marker) is shown based on the cryo-EM structure of the preS1(2–48)/NTCP WT complex. The 9–17 aa region in preS1 (N9 and H17 shown as a marker) is inserted in this TM1-TM8b tunnel. (D) Width of the hollow is shown in the MD analysis by the distance between the C_β_ atom of I88 and the C_ζ_ atom of Y146 (shown by blue dashed line). (E) Width of the tunnel region is shown in the MD analysis by the distance between the C_α_ atoms of L31 and that of Q264 (shown by black dashed line).
(PDF)

S4 Fig Representative structures during MD simulations. (A) PCA showing the structural distribution of preS1(2–48)/NTCP WT complex during MD simulations (center), and representative structures for the points a (left) and b (right) are illustrated. In the snapshots, the tunnel and hollow regions are focused, and the residues L31, Q264, I88, Y146, and F274 in NTCP are shown by spheres. The preS1 of the complex is shown by violet, with N9, H17, and W41 represented as sticks. (B) PCA showing the structural distribution of apo NTCP (F274S) during MD simulations (center), and representative structures for the points a (lower right), b (lower left), c (upper right) and d (upper left) are illustrated. In the snapshots, the tunnel and hollow regions are focused, and the residues L31, Q264, I88, Y146, and S274 are shown by spheres. The superimposed preS1 structure for the cryo-EM structure of preS1(2–48)/NTCP WT complex is shown as an eye guide (pink). The superimpose between the snapshot of apo NTCP and the cryo-EM structure is done using PyMOL alignment command. (C) PCA showing the structural distribution of apo NTCP (F274C) during MD simulations (center), and representative structures for the points a (lower right), b (upper left) and c (upper right) are illustrated. In the snapshots, the tunnel and hollow regions are focused, and the residues L31, Q264, I88, Y146, and C274 are shown by spheres. The superimposed preS1 structure for the cryo-EM structure of preS1(2–48)/NTCP WT complex is shown as an eye guide (pink). The superimpose between the snapshot of apo NTCP and the cryo-EM structure is done using PyMOL alignment command.
(PDF)

S1 Table Profile of NTCP mutants for capacity to support viral infection, preS1 binding, bile acid uptake, and cell surface expression. The numeric values for Fig 6A are shown.
(PDF)

S2 Table System setup details of MD simulations. The details of membrane-water systems for four systems prepared by CHARMM-GUI are shown.
(PDF)

S1 Data All the relevant raw data are shown. (XLSX)
