# Supplementary figures and images for: Structural mapping of NTCP distinguishes its dual functionality as a hepatitis B virus receptor and bile acid transporter
Source: PLoS Pathog. 2026 Jan 16;22(1):e1013824. doi: 10.1371/journal.ppat.1013824 (PMC12810916; doi:10.1371/journal.ppat.1013824)

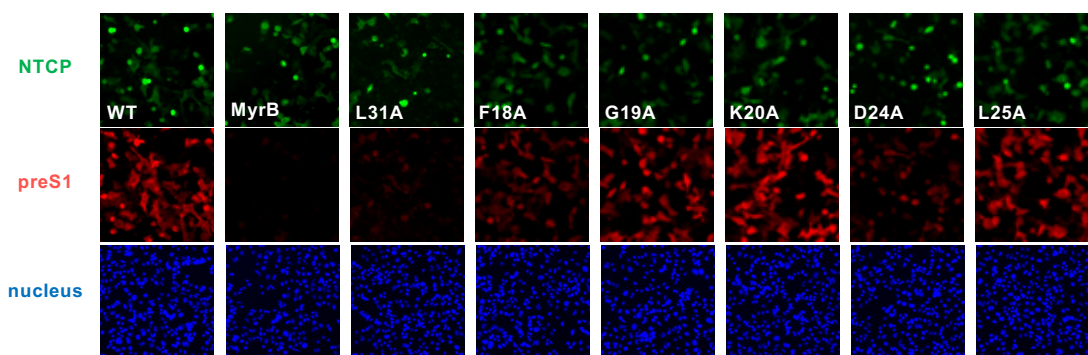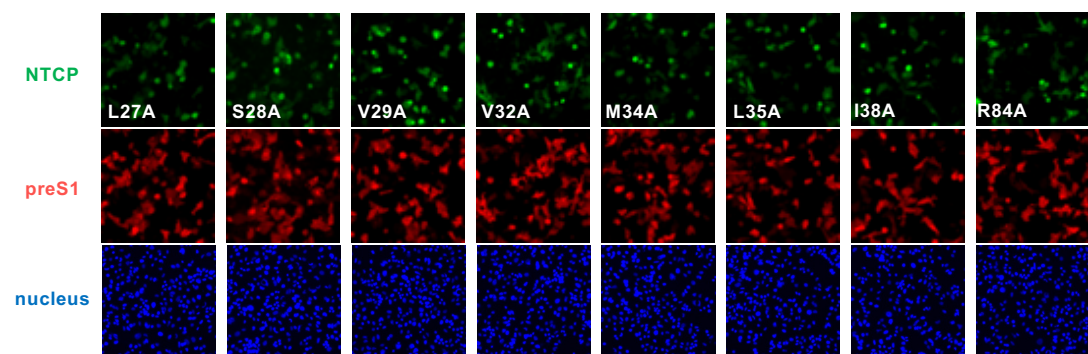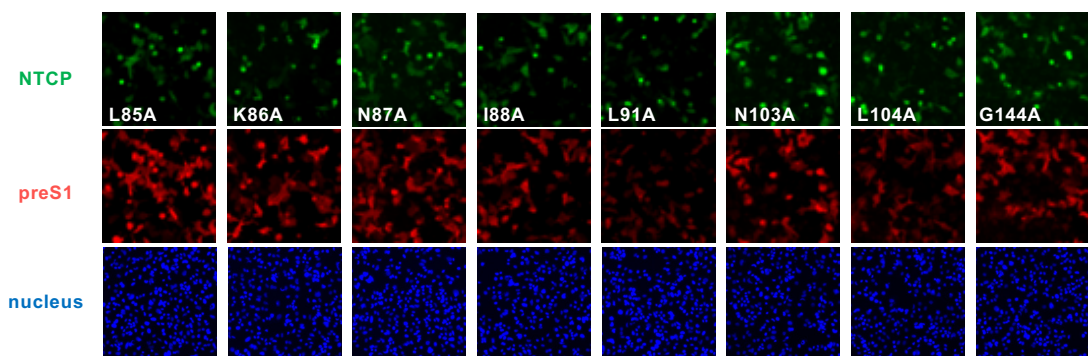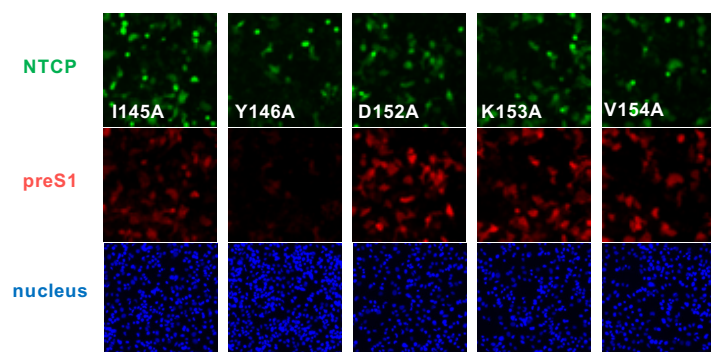

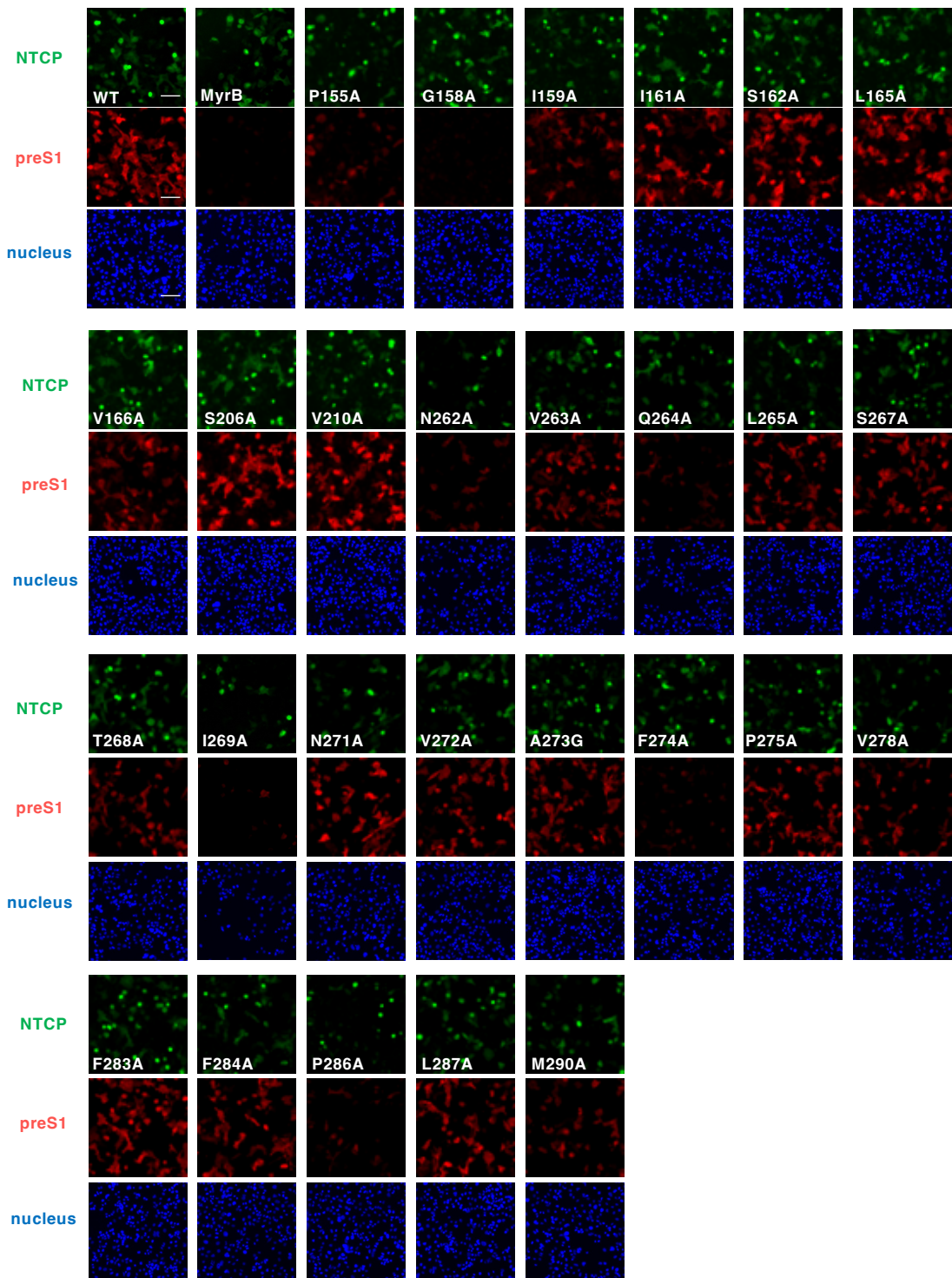

Supplement: S1 Fig — The enlarged immunofluorescence images of Fig 3B are shown. (PDF) [file ppat.1013824.s001.pdf]

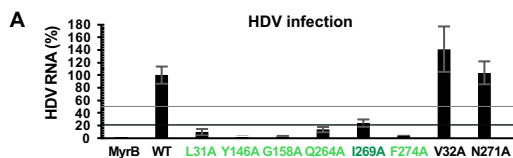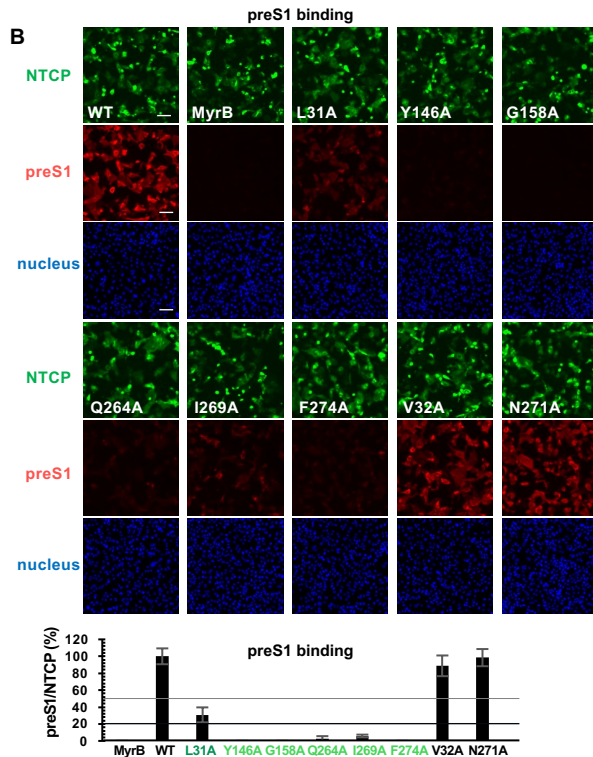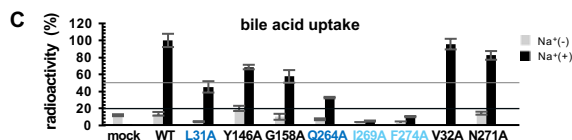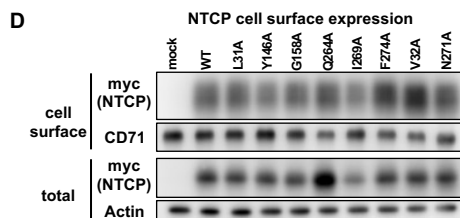

Supplement: S2 Fig — HDV infection (A), preS1 binding (B), bile acid uptake (C), and protein expression on cell surface and total fraction (D) were examined using Huh-7 cells overexpressing NTCP WT or its mutants, as described in Figs 2–5. (PDF) [file ppat.1013824.s002.pdf]

**A**

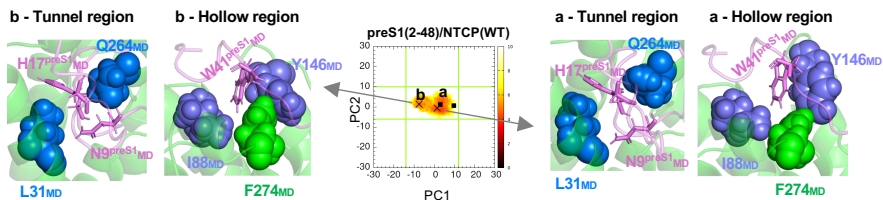

**B**

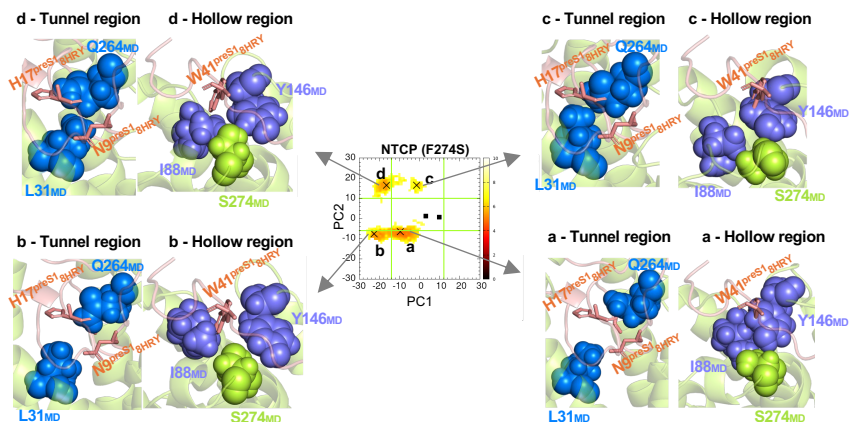

**C**

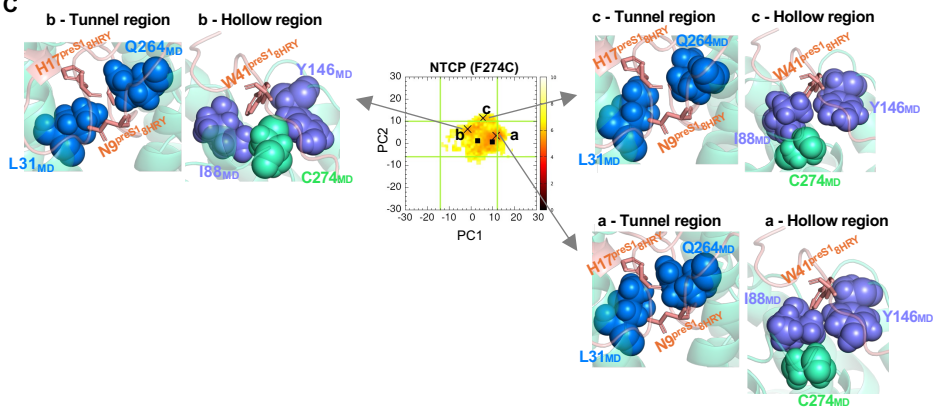

Supplement: S4 Fig — (A) PCA showing the structural distribution of preS1(2–48)/NTCP WT complex during MD simulations (center), and representative structures for the points a (left) and b (right) are illustrated. In the snapshots, the tunnel and hollow regions are focused, and the residues L31, Q264, I88, Y146, and F274 in NTCP are shown by spheres. The preS1 of the complex is shown by violet, with N9, H17, and W41 represented as sticks. (B) PCA showing the structural distribution of apo NTCP (F274S) during MD simulations (center), and representative structures for the points a (lower right), b (lower left), c (upper right) and d (upper left) are illustrated. In the snapshots, the tunnel and hollow regions are focused, and the residues L31, Q264, I88, Y146, and S274 are shown by spheres. The superimposed preS1 structure for the cryo-EM structure of preS1(2–48)/NTCP WT complex is shown as an eye guide (pink). The superimpose between the snapshot of apo NTCP and the cryo-EM structure is done using PyMOL alignment command. (C) PCA showing the structural distribution of apo NTCP (F274C) during MD simulations (center), and representative structures for the points a (lower right), b (upper left) and c (upper right) are illustrated. In the snapshots, the tunnel and hollow regions are focused, and the residues L31, Q264, I88, Y146, and C274 are shown by spheres. The superimposed preS1 structure for the cryo-EM structure of preS1(2–48)/NTCP WT complex is shown as an eye guide (pink). The superimpose between the snapshot of apo NTCP and the cryo-EM structure is done using PyMOL alignment command. (PDF) [file ppat.1013824.s004.pdf]
