# Supplementary material for: Structural mapping of NTCP distinguishes its dual functionality as a hepatitis B virus receptor and bile acid transporter
Source: PLoS Pathog. 2026 Jan 16;22(1):e1013824. doi: 10.1371/journal.ppat.1013824 (PMC12810916; doi:10.1371/journal.ppat.1013824)
Supplement: S3 Fig — (A) A membrane-water system for apo NTCP used in the MD simulations. NTCP is represented as green cartoon, membrane molecules are gray lines, and water, sodium, and chloride molecules are cyan, yellow, and green color spheres, respectively. (B) The outer-surface hollow formed by I88, Y146, and F274 is shown based on the cryo-EM structure of the preS1(2–48)/NTCP WT complex. W41 in preS1 is docked in this extracellular hollow. Green and pink cartoon indicate NTCP and preS1, respectively. (C) The tunnel region formed by TM1 (L31 as a marker) and TM8b (Q264 as a marker) is shown based on the cryo-EM structure of the preS1(2–48)/NTCP WT complex. The 9–17 aa region in preS1 (N9 and H17 shown as a marker) is inserted in this TM1-TM8b tunnel. (D) Width of the hollow is shown in the MD analysis by the distance between the Cβ atom of I88 and the Cζ atom of Y146 (shown by blue dashed line). (E) Width of the tunnel region is shown in the MD analysis by the distance between the Cα atoms of L31 and that of Q264 (shown by black dashed line). (PDF) [file ppat.1013824.s003.pdf]

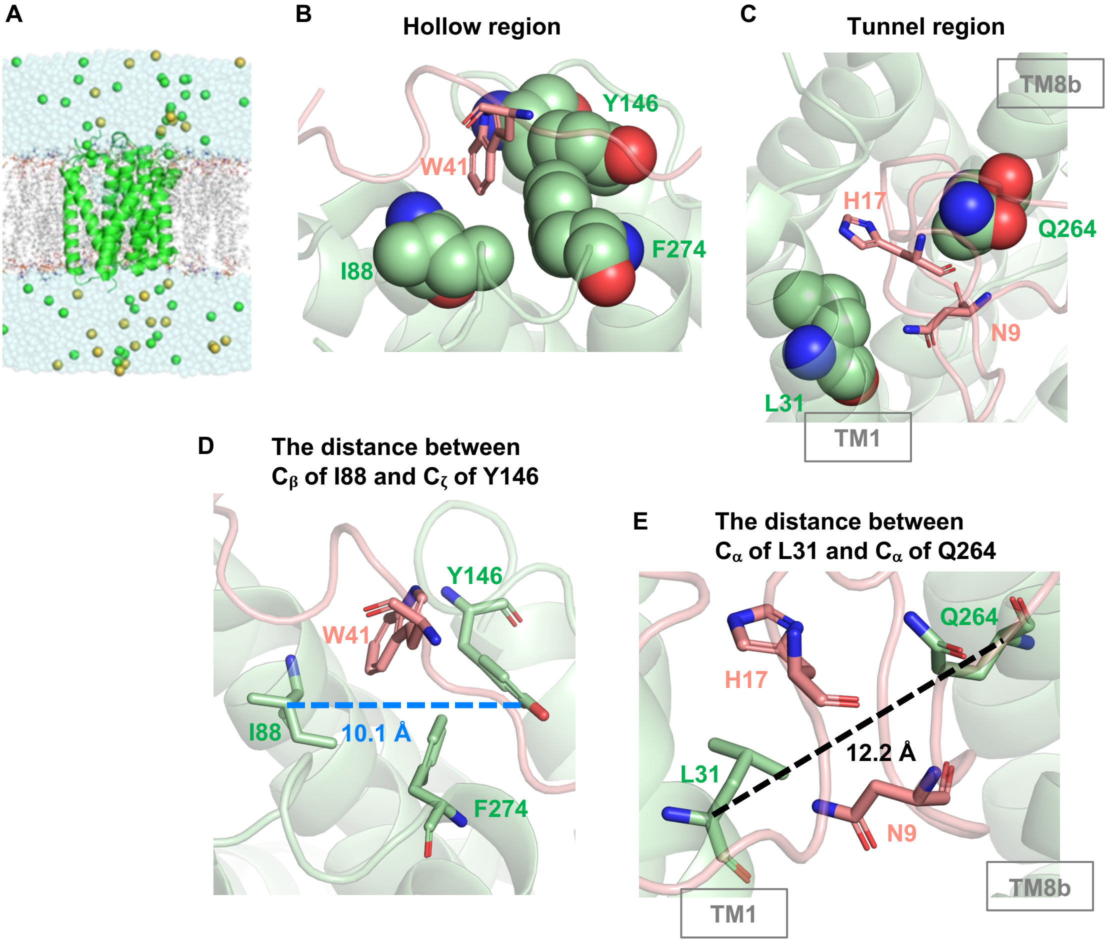

A
B
Hollow region
Y146
W41
I88
F274
C
Tunnel region
TM8b
H17
Q264
N9
L31
TM1
D
The distance between
C_β of I88 and C_ζ of Y146
Y146
W41
I88
10.1 Å
F274
E
The distance between
C_α of L31 and C_α of Q264
Q264
H17
12.2 Å
L31
N9
TM8b
TM1
