# Supplementary material for: Structural mapping of NTCP distinguishes its dual functionality as a hepatitis B virus receptor and bile acid transporter
Source: PLoS Pathog. 2026 Jan 16;22(1):e1013824. doi: 10.1371/journal.ppat.1013824 (PMC12810916; doi:10.1371/journal.ppat.1013824)
Supplement: S1 Table — The numeric values for Fig 6A are shown. (PDF) [file ppat.1013824.s005.pdf]

		extracellular			TM1									extracellular					TM3a
	WT	F18	G19	K20	D24	L25	L27	S28	V29	L31	V32	M34	L35	I38	R84	L85	K86	N87	I88	L91
virus infection	100	72	94	91	32	93	68	100	79	24	96	89	70	107	91	78	73	33	55	35
preS1 binding	100	62	102	105	43	100	103	97	95	19	102	100	95	90	96	103	99	80	81	42
bile acid uptake	100	66	74	133	18	82	87	82	112	47	124	53	43	79	60	53	72	66	58	35
cell surface expression	100	59	135	121	45	89	105	115	112	140	133	78	97	89	103	102	113	134	124	53

		TM3a		extracellular							TM5					TM6	
	WT	N103	L104	G144	I145	Y146	D152	K153	V154	P155	G158	I159	I161	S162	L165	V166	S206	V210
virus infection	100	86	68	111	100	2	119	96	52	44	9	79	121	47	58	83	102	81
preS1 binding	100	91	66	98	59	9	82	81	68	47	1	68	94	88	89	70	86	101
bile acid uptake	100	68	55	100	103	115	84	137	93	118	67	19	82	77	63	31	89	92
cell surface expression	100	122	69	81	87	97	137	131	117	105	127	103	139	129	130	143	116	111

		TM8										extracellular			TM9			
	WT	N262	V263	Q264	L265	S267	T268	I269	N271	V272	A273	F274	P275	V278	F283	F284	P286	L287	M290
virus infection	100	45	58	29	47	75	32	26	98	49	52	4	73	67	67	53	16	84	51
preS1 binding	100	45	91	6	104	97	71	10	117	102	87	2	85	71	108	99	31	102	74
bile acid uptake	100	22	67	50	58	63	82	2	90	18	93	2	63	72	75	63	17	60	58
cell surface expression	100	77	97	68	68	93	134	76	85	100	79	112	90	82	111	82	13	73	69
