# Supplementary material for: Structural mapping of NTCP distinguishes its dual functionality as a hepatitis B virus receptor and bile acid transporter
Source: PLoS Pathog. 2026 Jan 16;22(1):e1013824. doi: 10.1371/journal.ppat.1013824 (PMC12810916; doi:10.1371/journal.ppat.1013824)
Supplement: S2 Table — The details of membrane-water systems for four systems prepared by CHARMM-GUI are shown. (PDF) [file ppat.1013824.s006.pdf]

System	NTCP-2-48^{preS1}(WT)	apoNTCP(WT)	apoNTCP(F274S)	apoNTCP(F274C)
**system setup details of MD simulations**				
**Box dimensions (Å^3)**	70 x 70 x 108	70 x 70 x 104	70 x 70 x 104	70 x 70 x 104
**Total # of atoms**	48860	46951	46937	47208
**Total # of water molecules**	9944	9422	9421	9466
**Salt concentration**	150 mM NaCl	150mM NaCl	150 mM NaCl	150 mM NaCl
**Lipid composition (type, # of molecules)**	POPC, 100	POPC, 103	POPC, 103	POPC,104
